# On the Resuscitation of Apparently Dead Newborn by Laborde’s Method1Read at the thirty-second annual meeting of the Medical Association of Central New York, at Syracuse, October 17, 1899.

**Published:** 1900-01

**Authors:** Francis Eustace Fronczak

**Affiliations:** Buffalo, N.Y.


### ON THE RESUSCITATION OF APPARENTLY DEAD NEWBORN BY LABORDE'S METHOD.[1]

By FRANCIS EUSTACE FRONCZAK, A. M., M. D., Buffalo, N. Y.

ON JULY 5, 1892, Prof. Laborde communicated to the Paris Academy of Medicine a new method of resuscitating the apparently dead, by a method called by him, "the rhythmic traction of the tongue." This method, as we see by the name, consists in

1. Read at the thirty-second annual meeting of the Medical Association of Central New York, at Syracuse, October 17, 1899.

the rhythmic traction of the tongue by two fingers covered by ordinary cotton or a handkerchief. The traction being at the respiratory rate of 18-20 per minute in adult, a little more rapid in the new-born.

The author explains the success of this method by saying that the action results in a reflex irritation, which is referred to the respiratory center through the motions at the base of the tongue, the nerves which respond being the superior laryngeal, glosso-pharyngeal, the lingual and finally the phrenic. The method was first used exclusively, and even now is used by the majority of the profession in resuscitating those who stop breathing under chloroform, the drowned, the would-be suicides by hanging, and later has been used successfully in asphyxia neonatorum. I propose to quote several cases where this method gave gratifying results. It was some time before Laborde was able to arouse any attention for his method. For a long time he collected statistics and reports of cases bearing on this mode of reviving the apparently dead. The German obstetricians ridiculed the method and for a time it appeared that Laborde's labor would be in vain. Now, however, as we can see from quite a number of reported cases, the method is being brought more into vogue, and is gaining a well-deserved reputation and I believe is bound to stay.

As you know, by asphyxia neonatorum, we understand that condition where the newborn does not make any respiratory movements, notwithstanding the pulsation of the heart and of the cord more or less vigorously. We differentiate two degrees of apparent death of the newborn, the livid or lesser degree, where the skin is dusky, cyanotic, the cardiac and umbilical pulsations, though slow, are quite forcible, and reflex movements are easily produced by irritation of the skin; the pallid, or greater degree of aspyhxia, where the heart beats are very feeble and the pulsations of the cord is either almost or quite imperceptible. The child is pale and no reflex movement of any kind is possible.

It is outside my limits to speak of the etiology of this suspended animation of the newborn, sufficient for me to say that the essential cause of it, in my opinion, is not only the interference with placental circulation, but also various other causes, in my experience forceps being among these most common, and where the asphyxia is most prominent, this latter being due to the stoppage of return circulation from the brain of the child, with the resulting engorgement of the cerebral cortical vessels and rupture of these vessels into the brain tissue.

I shall only mention the other various methods of treatment of asphyxia neonatorum, so well known to you, the Hall, the Sylvester, the Schultze, the mouth to mouth insufflation, the chest compression, the Prochownik feet suspension, the Brown injection of brandy, the Cooke insertion of finger into rectum, and the many other things which have been proposed from time to time by various observers and real students of their surroundings, and the means of adapting them to their needs. As a rule, by far the greatest number use Schultze's method, which, we must admit, is most ingenious and feasible, as it answers the purpose in removing the foreign bodies from the respiratory tract, bringing about artificial respiration and most efficiently aids the circulation of the blood in the newborn. Yet this very commonly practised method has several things against its use. It is, in the first place, very tiresome to the person using it; secondly, it cannot be used where there is a fracture of the clavicle, as it may injure the pleura, or even penetrate the lung, though Schultze says that by proper use of this method there is no danger in using it in this fracture; third, it can hardly be used if there is a fracture of any of the extremities, either hand or leg, and we all know such fractures can occur in the practice of even the most experienced and careful obstetrician; fourth, this method does not give good results in prematurely born children; and fifth, those here present who practise among the poorer classes, know full well that in our practice we find rooms where the ceilings are so low that it is almost impossible to stand erect, much less to use Schultze's method of resuscitation of the newborn.

Schultze's method of swinging the child, requires a certain technique, which is seldom learned by the midwives; the fact is I have never seen a midwife perform these movements properly.

Now, Laborde's method does not have any of these faults which I have mentioned, but on the other hand has several points in its favor. The question may arise, can we resuscitate the newborn by the Laborde method as well as by the Schultze method? I believe there is no doubt in any one's mind here present, that in a mild degree of asphyxia neonatorum, where the sprinkling of the child with cold water will bring about the respiratory movement, Laborde's method will also give good results, but will the rhythmic traction of the tongue resuscitate a child apparently dead and in deep asphyxia, is a question which theoretically can be answered in the affirmative, and practically it has been proven to be so.

Schultze, himself, in an article in which he points to the mistakes made in the application of his method admits the feasibility of Laborde's method in mild and even in certain greater degrees of asphyxia, but he doubts if the French mode of resuscitating can be applied in very deep asphyxia, where there is no pulsation of the heart or cord, unless his (Schultze's) or some other method be used at the same time. Permit me, however, to quote a few cases in my practice, in which this theorised fear of Schultze's did not materialise, and Laborde's method held its own :

Case I.—Called by midwife to a multipara; prolapse of the arm; version under chloroform; leg pulled down; all well until head, which seemed to be almost immovable. The newborn in deep asphyxia. Schultze method used, no results; Prochownik's feet suspension—result negative; child put in warm bath and Laborde's rhythmic traction of the tongue at the rate of 25 per minute were made; after ten minutes child slowly revived.

Case II.—Multipara, transverse position; prolapse of cord; version; child born in deep asphyxia; sprinking with cold water and putting child in warm bath gives negative results; pulsation of heart not apparent. With Laborde's method, at almost every traction of the tongue there was slight respiratory movement, which ceased as soon as tractions were stopped. After several minutes gradually the child began to breathe.

Case III.—Forceps case; child delivered in deep asphyxia; Schultze method, insufflation, feet suspension, warm bath and cold water sprinkling—all in vain; traction of the tongue at the rate of about twenty per minute, continued for about twenty minutes, child gradually began to breathe irregularly and spasmodically and finally regular breathing ensued. Woman in labor for three days.

Case IV. —Tedious delivery, high forceps, male child in deep asphyxia; Laborde method, tractions at the rate of about twenty-two per minute, with child in warm bath; after ten minutes child resuscitated.

These, in short, are a few cases in which the results are very positive. In each case the nose and mouth were cleared of mucus. Let me repeat, the Laborde technique. As I have said before it consists in tractions of the tongue by two fingers wrapped in cotton or handkerchief, with child in warm bath. As in all other methods of resuscitation of the newborn, do not lose courage if after several minutes there are no results or signs of breathing. The tongue at first will give no resistance, after awhile it resists positively, soon we notice very mild respiratory movement, then all is quiet. In a short time the breathing is stronger and has a normal character and the child begins to cry, move, etc. Laborde's method is better than Schultze's because the child does not become chilled, being all the time in warm bath. We can notice the beating of the heart, do not tire ourselves as easily as in Schultze's method and can use it in cases where the latter mode of resuscitation is impossible.

BIBLIOGRAPHY.

1. Laborde: "Du procédé des tractions rhythmées de la langue dans les diverses asphyxies." Semaine Médicale, 1892, pp. 266 and 463.

2. Laborde: Semaine Médicale, December 5, 1894.

3. Erskine: Med. and Surg. Rep., 1895, LXXII., 525.

4. Russell: Therap. Gaz., August 15, 1895.

5. Cameron: Montreal Med. Jour., 1895, Volume XXII, No. 12

6. Saks: "Przyczynek do cucenia pozornie zmarych noworodków za pomoca metody Labordéa." Medycyna, August 5, 1899, T. XXVII., No. 31, p. 732.

7. Ludwig Knapf: "Erfahrung über Laborde's rhythmisches Zungentraktionen nebst einigen Bemerkungen über Verletzungen durch diese und andere Wiederbelebungs methoden." Centralblatt für Gynäkol., 1896, No. 28.

8. Sterling: Medycyna, 1894, p. 225.

9. B. S. Schultze: "Ueber die beim Scheintod Neugeborener vorliegenden Indicationen." Centralblatt für Gynäkol., 1896, No. 37.

508 Fillmore Avenue.

DISCUSSION.

Dr. Sears, of Syracuse.—I was very much interested in Dr. Fronczak's paper and I think there are some very good points brought out there. The fact of putting the child in the warm bath while you are performing your other methods of resuscitation is an excellent idea in my experience, and any method which will allow you to do that is preferable to any method, I think, which will not allow you to do that. In speaking of forceps as being a cause perhaps of the asphyxia, I think that is very true. The only case that I have ever seen with a strong heart beat and yet without any respiration, was a case of prolonged forceps. I was called some to or three years ago with Dr. B. He had been at work a long time with the forceps in this case, and after I arrived it took a long time, until I was nearly exhausted, before we could deliver the child. In that child, I think the heart beat strong for nearly twenty minutes, and yet we could get no respirations with every method that we could try and the child died. The real actual cause I don't know, but it was the only case I have ever seen where, with good strong heart beats, you could not get any respiration.

There is another method which I have used. I don't know but others have used it also. In cases where a child will breathe fairly well and yet will have a blue condition, cold extremities and feet and hands will become blue at intervals and doesn't revive properly. In this case, I have used warm saline, with a few drops of whiskey or brandy in the rectum and they respond very nicely. Four or five drops of brandy, with a teaspoonful of warm saline solution in the rectum will revive a baby very nicely in those cases where you have feeble respirations and cold extremities and without proper circulation.

I am greatly indebted to Dr. Fronczak for this paper, as this method is comparatively new to me. I have not used the methods that he speaks of.

Dr. Gabb.—I would like to know just what the process is to produce this condition. I have seen some children resuscitated and I have seen others die, but just what the *modus operandi* of this is, I am willing to say I don't understand.

Dr. Fronczak.—In answer to the first speaker, I would say that I have had two or three cases where the heart beat could be felt and seen and still the child did not revive. This contraction of the tongue, I will answer the Doctor, consists in taking the tongue and simply pulling it up and down at the rate of about twenty times per minute. That is the only thing he does to it. It seems to be so simple that I am surprised that the doctors expressed their idea of this being a new thing. I believe I am one of the first to use it in these cases in Buffalo. I had a case in the Penitentiary and I told my assistant to do that. He laughed at me. He said, "What good will that do?" It seemed to do some good, because the child revived.

